# Editorial: NK cells in the tumor microenvironment: immunosuppressive mechanisms and therapeutic opportunities

**DOI:** 10.3389/fimmu.2025.1606236

**Published:** 2025-04-23

**Authors:** Jose M. Ayuso, Jorrit De Waele

**Affiliations:** ^1^ Department of Dermatology, University of Wisconsin-Madison, Madison, WI, United States; ^2^ Center for Oncological Research (CORE), Integrated Personalized and Precision Oncology Network (IPPON), University of Antwerp, Antwerp, Belgium

**Keywords:** Natural Killer cells, tumor microenvironment, cancer immunotherapy, cell adoptive therapy, solid tumors

This Research Topic discusses the role of cellular and molecular immunosuppressive mechanisms that affect Natural killer (NK) cell response. In their review, Álvarez-Carrasco et al. provide an accessible introduction to NK cell immunobiology. NK cells are immune cells with cytolytic cells that target virus-infected or aberrant cells. Once activated, NK cells induce cytotoxicity by secreting pro-apoptotic proteins like TRAIL or injecting cytotoxic proteins like granzymes or perforins into the target cell. NK cells also secrete pro-inflammatory cytokines, contributing to immune recruitment to the affected tissue. Thus, NK cells represent an appealing cell source for adoptive cell therapy. However, previous studies have shown that solid tumors generate an immunosuppressive tumor microenvironment (TME) that blunts NK cell function.

Jiang et al. review cellular and molecular mechanisms controlling NK cell function and their implications for cancer immunotherapy. First, Jiang et al. provide an introduction detailing the activation and inhibition mechanisms controlling NK cell activity. In this context, NK cells possess a repertoire of activatory and inhibiting receptors that determine whether the NK cell lyses the target cell. The authors also discuss how other immune cells like macrophages, dendritic cells, or T cells interact with NK cells in the TME. Next, Jiang et al. review clinical trials using NK cells against solid and hematological malignancies. They describe relevant strategies to improve NK cell efficacy in the TME including the use of chimeric antigen receptors (CARs), cytokine supplementation, or combination with monoclonal antibodies to increase NK cell specificity and cytotoxicity. They also describe the use of immune checkpoint inhibitors like anti-PD1 or anti-TIGIT antibodies, as well as the development of NK cell engagers, which are based on bi-, tri-, or tetra-specific engineered antibodies that simultaneously target tumor and NK cells to promote NK cell recruitment and activation. This review provides an updated discussion of the state-of-the-art in NK cell immunotherapy discussing previous and ongoing strategies in NK cell immunotherapy.

NK cells are known for their capacity to elicit antibody-dependent cell cytotoxicity (ADCC), which allows them to destroy tumor cells coated by antibodies. ADCC contributes to the success of monoclonal antibodies like trastuzumab or pertuzumab, which target HER2+ cancer cells. However, how the TME affects ADCC still needs to be better defined. In their Original Research article, Batalha et al. leveraged a human 3D model of breast cancer for the study of anti-HER2 antibodies and their effects on NK cells. The authors generated multicellular spheroids using HER2-expressing cell lines and then embedded them in alginate microspheres in combination with PBMCs or NK cells extracted from healthy donors. They demonstrated the capacity of their model to visualize NK cell-mediated ADCC by using trastuzumab and pertuzumab against HER2+ breast cancer aggregates. Interestingly, they observed that the use of monoclonal antibodies like trastuzumab and pertuzumab led to progressive downregulation of the Fc receptor CD16 on NK cells, which is critical for antibody recognition and ADCC. Additionally, they also showed that the use of these antibodies selectively depleted CD56^dim^/CD16^+^ NK cells (known as cytotoxic NK cells) and increased CD56^bright^/CD16^-^ NK cells (which do not exhibit cytotoxic function and are abundant in lymphoid tissues), which would contribute to progressive loss of ADCC-dependent cytotoxicity against HER2^+^ tumor cells.

Oncolytic viruses are garnering interest as novel immunotherapeutic modality in cancer. Reovirus 3 strain Dearing is one of the viruses under clinical investigation, showing selective lysis of RAS-transformed cells and activation of the immune system. In their Original Research, Khaleafi et al. investigated the effects of reovirus on tumor-expressed ligands to a major activating NK cell receptor, NKG2D. They demonstrated a reduced expression of MICA, MICB, ULBP2, and ULBP3 in reovirus-infected cancer cells. Unaltered gene transcription, protein degradation and protein shedding could not explain this reduction, nor interference by any of the 10 viral genes that constitute the reovirus genome. While not explored in detail, the authors postulate that reovirus modulated protein translation of NKG2D ligands in cancer cells. Next, Khaleafi et al. showed that the downregulated expression of NKG2D ligands resulted in reduced binding of NKG2D. This negatively affected the cytotoxicity of NK cells towards reovirus-infected cancer cells, as well as their proliferation and IFN-γ production. Overall, their research demonstrated that reovirus alter NK cell evasion mechanisms. This warrants further investigation of other receptor-ligand pathways which could lead to promising combination approaches for oncolytic virotherapy.

Labeled as the guardian of the genome, p53 is the most commonly mutated gene in cancer. In their Review Article, Wang et al. discuss the multifaceted role of p53 in tumor immune surveillance by NK cells. While there is still uncovered ground regarding p53 in NK cells, the authors discuss how the protein regulates cell cycle apoptosis in NK cells, but as transcription factor is also involved in other processes aiding NK cell activation and response to the TME. On tumor cells, p53 modulates the expression of NK cell ligands favoring their anti-tumor functioning. It is also involved in cell death mechanisms, autophagy and metabolism. Next, the role of p53 in other stromal cells is discussed, as well as its effects on the extracellular proteome in the TME. Intriguingly, reactivation of p53 can modulate the TME in a way that suppresses NK cell functions, such as clearance of senescent tumor cells. As *TP53* is often mutated, the authors describe the associated negative effects on NK cells and their function. Wang et al. conclude by debating p53 as a potential target for NK cell therapies such as using p53-suppressor MDM2, which has shown beneficial effects in combination with both immune checkpoint inhibitors and CAR NK cells. The review offers insights into the direct/indirect relationship of p53, in its different forms, to NK cell-mediated tumor surveillance in the view of advancing NK cell-based immunotherapy through p53-based combinations.

In conclusion, the articles within this Research Topic cover basic concepts in NK cell biology and immunotherapy as well as more specialized areas within the NK cell-cancer field, highlighting the potential of NK cells in antitumor immunity and the discernible role of the TME in the view of advancing NK cell cancer immunotherapy.

